# Supplementary figures and images for: Emulsion Properties during Microencapsulation of Cannabis Oil Based on Protein and Sucrose Esters as Emulsifiers: Stability and Rheological Behavior
Source: Foods. 2022 Dec 5;11(23):3923. doi: 10.3390/foods11233923 (PMC9735479; doi:10.3390/foods11233923)

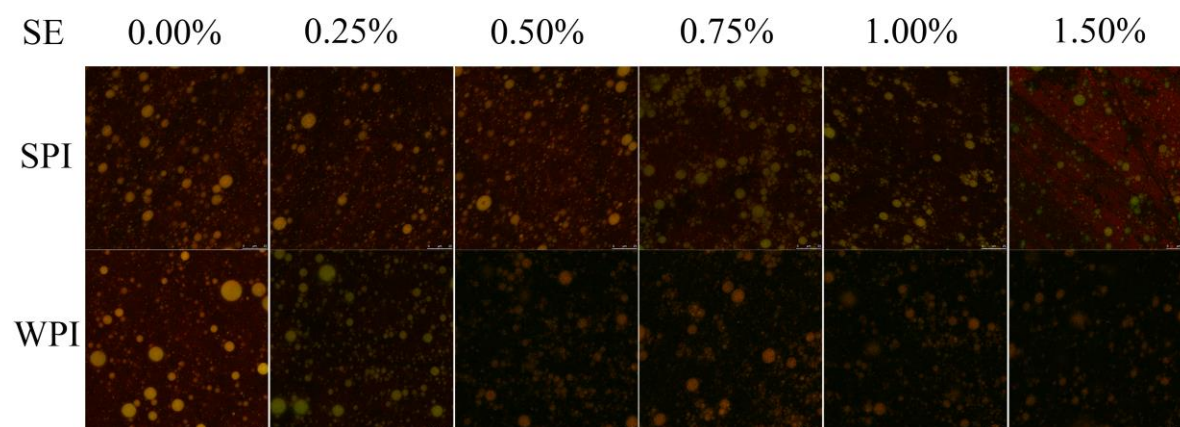

**Figure S1.** Microstructure of emulsion at different concentrations of SE (0.00–1.50%).

Supplement: Supplementary file 1 [file foods-11-03923-s001.zip › foods-1988388-supplementary.pdf]
